# Molecular and epidemiological surveillance of *Plasmodium* spp. during a mortality event affecting Humboldt penguins (*Spheniscus humboldti*) at a zoo in the UK^☆^

**DOI:** 10.1016/j.ijppaw.2022.06.010

**Published:** 2022-07-05

**Authors:** Merit González-Olvera, Arturo Hernandez-Colina, Tanja Himmel, Lindsay Eckley, Javier Lopez, Julian Chantrey, Matthew Baylis, Andrew P. Jackson

**Affiliations:** a Institute of Infection, Veterinary and Ecological Sciences, University of Liverpool, Ic2 Liverpool Science Park, 146 Brownlow Hill, Liverpool, L3 5RF, United Kingdom; b Institute of Pathology, University of Veterinary Medicine Vienna, Veterinaerplatz 1, 1210, Vienna, Austria; c North of England Zoological Society (Chester Zoo), Caughall Road, Chester, CH2 1LH, United Kingdom

**Keywords:** Avian malaria, Culex, Mosquito, Penguin, *Plasmodium*, *Spheniscus*, Zoo

## Abstract

In 2017, a mortality event affected Humboldt penguins at Chester Zoo (UK), which coincided with the diagnosis of avian malaria (AM) in some birds. AM is found worldwide wherever a competent mosquito vector is present, but the disease is particularly severe in penguins and other species that originate from non-endemic regions. To better understand the role of AM and manage its threat to penguin collections, *Plasmodium* was surveyed through PCR at Chester Zoo in mosquitoes, penguins, and dead free-living wild birds during and around the mortality event. Additional sequences were obtained from penguin fatalities from four other UK zoological collections. All sequences were integrated into phylogenetic analyses to determine parasite species and lineages. In total, 753/6459 positive mosquitoes were recorded (11.7% prevalence), reaching a weekly peak of 30% prevalence in mid-summer. Among penguin fatalities at Chester Zoo, several penguins presented signs and lesions compatible with AM; nevertheless, exoerythrocytic meronts were identified in only one case and *Plasmodium* spp. was identified in 5/22 birds. Phylogenetic analysis revealed at least five parasite *cytb* lineages of three *Plasmodium* species (*P. matutinum, P. relictum* and *P. vaughani*) circulating in mosquitoes at Chester Zoo; however, infections in free-living wild birds and penguins were only from *P. matutinum*. *Plasmodium matutinum* was confirmed as the cause of death of one penguin and was highly suspected to be the cause of death of another three. The lineage LINN1 was associated with 4/5 penguin infections. AM had a key role in the penguin multicausal mortality event. Understanding the risk of AM to penguin collections at Chester Zoo and elsewhere requires long-term surveillance to examine the association between *Plasmodium* infection and penguin mortality and the variability in parasite virulence. Surveillance of *Plasmodium* spp. in mosquitoes and local birds provides information about the parasite's transmission cycle locally, and could warn about infection risks to species of interest, which is essential for efficient disease control and prevention.

## Highlights

• *Plasmodium* infection in penguins, mosquito and wild birds is described.
• Humboldt penguin mortality due to *P. matutinum* was recorded for the first time.
• One of three *Plasmodium* spp. found in mosquitoes infected penguins and wild birds.
• Detecting *Plasmodium* infections is challenging despite pathological evidence.

## Graphical abstract

**Figure:**
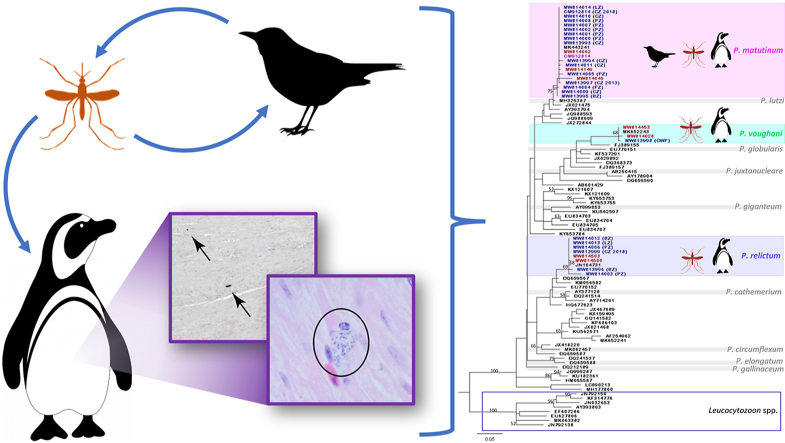

## 1 Introduction

Malaria parasites (*Plasmodium* spp.) are endemic among bird populations worldwide, where they are transmitted by mosquitoes, but are infrequently associated with host mortality or serious illness. Avian malaria is typically considered a subclinical infection that only leads to significant mortality when introduced into bird communities lacking prior exposure to the parasite, either due to biogeographical isolation or cold climate (Foster et al., 2007; Lapointe et al., 2012). For instance, native birds of New Zealand have been disproportionately affected by *Plasmodium relictum* introduced with European bird species (Schoener et al., 2014; Niebuhr et al., 2016), while the introduction of avian malaria into Hawaii has contributed to the extinction of native honeycreepers (Carduelinae) at altitudes low enough to maintain the mosquito vector (Lapointe et al., 2012; Paxton et al., 2016).

Penguins have a high susceptibility to serious or fatal avian malaria presentation, possibly associated to a lack of exposure to the parasite, since they inhabit environments too cold to sustain mosquitoes (Foster et al., 2007; Lapointe et al., 2012). The disease is cited as a leading cause of death among ex-situ managed penguins in zoos worldwide, with mortality ranging between 10 and 83% (Graczyk et al., 1995; Grilo et al., 2016). In the UK, between 1999 and 2018, 75 avian malaria outbreaks (38 suspected, 37 confirmed) affected the penguin colonies of 18 zoos (Hernandez-Colina et al., 2021a). *Plasmodium* spp. are also emerging pathogens of wild penguins; for instance, yellow-eyed penguins (*Megadyptes antipodes*) in New Zealand (Alley et al., 2019). This raises concerns for their conservation and, specifically, the possibility that increasing global temperatures could result in avian malaria reaching penguin colonies that have been historically free from the parasite (Garamszegi, 2011).

While avian malaria undoubtedly poses its greatest threat to bird populations historically isolated from disease, the impact of infection is now understood to scale continuously from asymptomatic to lethal, and apparently subclinical infections have subtle but significant effects on host fitness (Alley et al., 2019). Ashgar et al. (2015) directly determined infection status among wild great reed warblers (*Acrocephalus arundinaceus*) by PCR and showed that low-level chronic malaria infection reduced longevity and fecundity (Asghar et al., 2015), while Knowles et al. (2010) showed that medicating wild blue tits (*Cyanistes caeruleus*) with the anti-malarial drug Atovaquone increased fecundity and offspring survival (Knowles et al., 2010).

This variation in disease impact is caused by multiple factors, not least host species (Palinauskas et al., 2008). However, parasite species is a major determining factor, and some species, such as *P. relictum* and *P. elongatum*, are considered particularly virulent pathogens with worldwide distribution (Sallaberry-Pincheira et al., 2015; Vanstreels et al., 2019). Overall, there are over 60 *Plasmodium* morphospecies described in birds, but more than 1500 genetic lineages that are not associated with a described species (Outlaw and Ricklefs, 2014) indicating that phylogenetic diversity runs far ahead of established taxonomy. Precisely how disease impact varies among these different parasites is not known, and the genetic causes of interspecific variation in virulence and pathogenesis are only beginning to be explored. Videvall et al. (2020) observed that high- and low-virulence strains of *P. relictum* cause up to 87% and <1% parasitaemia respectively in Eurasian siskins (*Spinus spinus*) and that this is associated with significantly different gene expression profiles in the parasites but not the hosts.

Although the genetic backgrounds of both host and parasite are clearly important, the precise factors that determine the occurrence of disease or its severity are not completely understood (Lapointe et al., 2012; Knowles et al., 2011). When avian malaria affects a penguin colony, preventive measures and treatment typically have inconsistent efficacy, as the colony could recover favourably or succumb to the disease (Hernandez-Colina et al., 2021a; Vanstreels et al., 2014). Disease may be fulminant, when individuals succumb acutely and die before parasitaemia is evident in the blood; in these cases, diagnosis by blood smears is not possible and molecular assays also frequently fail to detect it (Grilo et al., 2016; Stidworthy and Denk, 2018). Likewise, histological examination and PCR may also fail to evidence the parasites from highly suspected avian malaria casualties (Spottiswoode et al., 2020).

In early September 2017, the Chester Zoo (Cheshire, U.K.) Humboldt penguin (*Spheniscus humboldti*) colony was temporarily relocated to an indoor housing facility while the outdoor exhibit was refurbished. Precautions were taken to avoid the entrance of mosquitoes from windows and small gaps. A few weeks after the move, mortality presented in the colony and although not previously observed at Chester Zoo, avian malaria was suspected and *Plasmodium* was identified in some of the penguin fatalities. The presence of *Plasmodium* was monitored among penguins, free-living wild birds and mosquitoes around and during the mortality event to assess the contribution of avian malaria and to evaluate its on-going risk to the collection. A description of the malaria epidemiology and pathology observed at Chester Zoo is presented along with a molecular analysis of parasite prevalence over a seven-month period. Clustering and phylogenetic analysis is used to identify the *Plasmodium* species responsible for penguin infections and to examine genetic variation among the circulating parasites.

## 2 Materials and methods

### 2.1 Study site

Penguin, dead free-living wild birds and mosquito samples were collected at Chester Zoo (Upton by Chester, Cheshire, UK (53°13′21.60″ N −2°53′1.79″ W), a 51ha zoological garden housing over 21,000 animals (1912 birds) belonging to more than 500 species (154 bird species). The zoo occupies a peri-urban site, surrounded on three sides by mixed farmland and woodland. The Humboldt penguin colony is typically housed fully outdoors, but between September and December 2017, they were moved indoors during a period of refurbishment.

### 2.2 Penguin pathological examination

All penguins that died between September 2017 and January 2018 were subjected to a full necropsy. Samples from parenchymal organs typically implicated in *Plasmodium* infection (heart, lung, liver, spleen, kidneys and brain) and from tissues with gross lesions, were fixed in 10% buffered formalin for 24–48 h and subsequently embedded in paraffin wax and submitted for histopathological examination. Tissues were inspected under light microscopy after paraffin-embedded tissue sections (5 μm) were stained with haematoxylin and eosin (HE). Additional embedded sections were used for chromogenic in situ hybridization (CISH) (Himmel et al., 2020).

### 2.3 Avian malaria sampling in birds

Samples from at least three organs typically affected by avian malaria were taken from all but four penguins that died during the mortality event. Likewise, samples from brain and liver were taken from free-living wild birds found dead on the zoo premises. DNA was extracted from blood and organ samples and tested by PCR. Surplus blood from penguin samples obtained as part of diagnostic procedures during the seven month period of the study were also analysed in this study. Three thin blood smears were prepared per bird. The smears were fixed with absolute methanol and stained with Giemsa. Each smear was screened for 30 min under high magnification (1000 X) as suggested by Valkiūnas (2005).

### 2.4 Additional penguin samples

To supplement the parasites obtained directly from penguin infections, and thereby provide context to the *Plasmodium* spp. discovered at Chester Zoo, we examined 20 penguin liver samples from previously histopathologically confirmed avian malaria fatalities at five other UK collections. These sites were ZSL London Zoo (*S. humboldti*; n = 2), Paignton Zoo and Animal Wildlife Park (*S. humboldti*; n = 8, *Eudyptes chrysolophus*; n = 5), Blackpool Zoo (*S. magellanicus*; n = 3) Cotswold Wildlife Park (*S. humboldti*; n = 1) and Folly Farm (*S. humboldti*; n = 1). Paington Zoo samples were obtained directly from their collections; the rest of the samples came through the International Zoo Veterinary Group, where they were submitted for diagnostic purposes. We also continued testing penguin blood (n = 19) and organ samples (n = 1) from Chester Zoo in 2018, and archived, frozen penguin organs from Chester Zoo collected in previous years: 2011 (n = 2), 2012 (n = 2), 2013 (n = 1), 2014 (n = 1), 2015 (n = 2), and 2016 (n = 3).

### 2.5 Chromogenic in situ hybridization (CISH)

For detecting *Plasmodium* parasites in tissue sections, CISH was performed using a previously designed digoxigenin-labeled oligonucleotide probe (sequence 5′-TTTAATAACTCGTTATATATATCAGTGTAGCAC-3′) and established protocols (Dinhopl et al., 2011). The genus-specific probe targets the 18S ribosomal RNA of avian *Plasmodium* spp. and has proven successful for detecting avian malaria parasites in penguins and other bird species (Igūnas et al., 2016; Himmel et al., 2020). In brief, 1–2 μm tissue sections were deparaffinized, rehydrated in an ascending series of ethanol and distilled water, and subjected to proteolytic treatment with Proteinase K (Roche, Basel, Switzerland) 3 μg/ml in 0.5 M Tris–HCl buffered saline for 40 min at 37 °C. After rinsing in distilled water, tissue sections were dehydrated in ethanol, air-dried and incubated overnight at 40 °C with hybridization solution containing 11 μl distilled water, 20 μl 20 × saline-sodium citrate (SSC) buffer, 50 μl formamide, 5 μl herring sperm, 2 μl 50 × Denhardt's solution, 10 μl dextran sulphate (50%, w/v) and 1 ng probe per 100 μl. The following day, sections were subjected to stringent washes in 2 × , 1 × and 0.1 × SSC buffer 10 min each and blocked with normal goat serum and 10% Triton X-100 for 30 min before incubation with anti-digoxigenin-AP Fab-fragments (Roche) at 1:200 for 1 h at room temperature. For chromogenic detection, sections were washed and covered with colour development substrates NBT/BCIP (nitro-blue tetrazolium chloride/5-bromo-4-chloro-3′- indolyphosphate p-toluidine salt, Roche) mixed with levamisole in 0.1 M TBS (pH 9.5) for 40 min in a dark, humid chamber. After terminating enzymatic reactions by washing in Tris–EDTA buffer (pH 8.0) for 10 min, sections were counterstained with haematoxylin and mounted using Aquatex (Merck Millipore) and coverslips.

### 2.6 Mosquito trapping

**Fig. 1:**
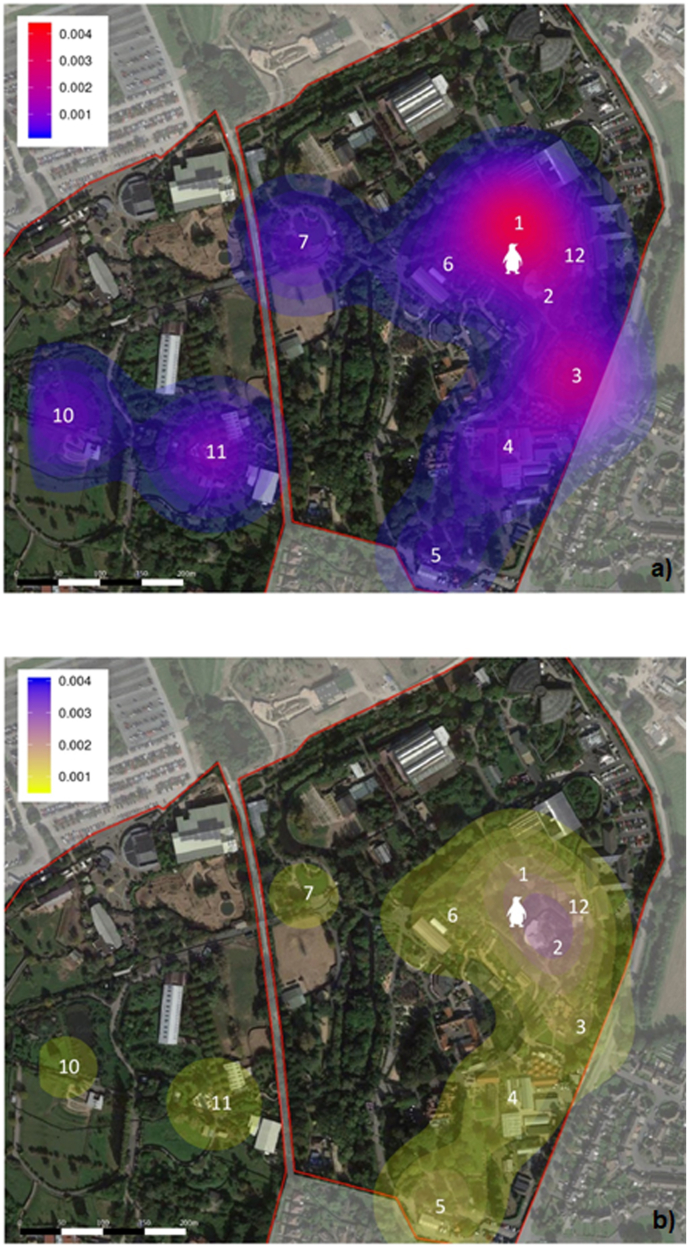
**Mosquito abundance and *Plasmodium* prevalence compared at ten sampling sites across Chester Zoo**. The Chester Zoo site (zoo perimeter is outlined) overlaid with a heat map of total mosquito numbers trapped at 10 sampling sites. Locations of traps are indicated by numbers 1–7, 10–12 inclusive. The location of the penguin exhibit in 2017 is indicated by a penguin symbol. **a.** Mosquito abundance. **b.***Plasmodium* prevalence in trapped mosquitoes. Heat maps were generated using Heatmapper with a Gaussian radius multiplier of 1.

Adult mosquitoes were captured in the zoo grounds using two kinds of trap, the BG-Mosquitaire trap and the CDC-Gravid trap model 1712, which were selected for their efficiency at capturing *Culex pipiens* (Reiter, 1983; Hernandez-Colina et al., 2021b). Ten sampling sites were established in or near bird enclosures at the zoo to trap adult mosquitoes, as shown in Fig. 1; they were defined as 30 m diameter areas containing one trap of each kind. When possible, the traps were protected from direct sunlight, artificial lighting, wind, and rain, and were sited near vegetation and water bodies. Traps were at least 10 m apart to avoid interference among them. Zoo staff was consulted prior to placing the traps to confirm that each site had easy access for sampling and electricity supply, while ensuring that traps did not represent a hazard for animals or visitors.

Trapping was carried out on two consecutive days per week from May to November 2017. Each week, on the first day the collection nets of BG-Mosquitaire traps were replaced and the CDC-Gravid traps were prepared; then, on the second day, the nets from the BG-Mosquitaire traps were again replaced and the nets from the CDC-Gravid traps were collected. Thus, two samples were collected from the BG-Mosquitaire traps per week, after six days and after 24 h respectively, while the CDC-Gravid traps were emptied once per week after 24 h. After collection, the nets were transported to the laboratory in an ice box and frozen at −20 °C on arrival.

### 2.7 Mosquito identification

All trapped mosquitoes were identified by individual observation under a stereoscope and following identification keys (Cranston et al., 1987; Becker et al., 2020). Mosquitoes were placed on a chill table at −16 °C during the process and stored in individual reaction tubes at −20 °C until further analysis. Some damaged mosquitoes were partially identified to genus or family level. Morphological distinction between *Cx. pipiens* and *Cx. torrentium* is unreliable. To distinguish between these two species, the conventional PCR and digestion enzymes protocol developed by Hesson et al. (2010) was used.

### 2.8 DNA extraction

Total DNA was extracted from each mosquito, from free-living wild bird organs and from blood and organ samples collected from penguins. DNA was extracted from mosquitoes using either an E.Z.N.A.® Tissue DNA Kit (Omega Bio-Tek) (n = 2072) according to the manufacturer's instructions, or the Livak DNA extraction protocol (Livak, 1984) (n = 4790). For birds' organs and blood, DNA extractions were performed using DNeasy kits (Qiagen) according to the manufacturer's instructions. Since the blood extracts produced a smaller DNA yield, the elution buffer volume was reduced to 50 μl.

### 2.9 Polymerase chain reaction

DNA extracted from mosquitoes, bird organs and blood samples were tested for *Plasmodium* spp. by nested PCR using the method described by Hellgren et al. (2004), which amplifies a 479-bp fragment of the cytochrome *b* (*cytb*) gene. For the first part of the PCR, each reaction included 1 μl of DNA template, 1 μl of forward primer HaemNF1, 1 μl of reverse primer HaemNR3, 10 μl of MyTaq™ Red Mix PCR mastermix, 1 μl of BSA and 6 μl of nuclease free water, to reach a final volume of 20 μl. The PCR profile was 22 cycles at 94 °C for 3 min, 94 °C for 30 s, 50 °C for 30 s, 72 °C for 45 s, followed by extension at 72 °C for 10 min. The second part was performed using 2 μl of PCR product from the previous reaction as template, 1 μl of forward primer HaemF, 1 μl of reverse primer HaemR2, 10 μl of MyTaq™ Red Mix PCR mastermix, 1 μl of BSA and 5 μl of nuclease free water, to reach a final volume of 20 μl. The profile for the second part was 36 cycles at 94 °C for 3 min, 94 °C for 30 s, 50 °C for 30 s, 72 °C for 45 s, followed by extension at 72 °C for 10 min. The amplicons were visualised on a 1.5% agarose gel stained using the SYBR Safe DNA gel satin (Thermo Fisher Scientific). Genomic DNA from *P. berghei* ANKA (rodent malaria parasite) was used as a positive control and molecular grade water was used as a negative control on every occasion. Mosquito DNA samples (2–4) were pooled per well, plus 8 negative controls and 4 positive controls per plate. In the event of a PCR-positive well, all individual samples from the well were then tested individually. Bird organ and blood samples were tested individually, adding a positive and negative control every ten samples. Mosquitoes from the 20th of July (n = 403) were contaminated while processing and therefore were not included in prevalence and further analysis.

### 2.10 DNA sequencing

Positive PCR reactions produced a ∼470 bp amplicon, each of which was sequenced using the Sanger dideoxy method from the 3' direction (Source Bioscience, Nottingham, U.K.). Poor quality amplicon sequences that contained dubious or ambiguous bases, or became too short (i.e. <380 bp) after the removal of such bases, were excluded.

### 2.11 Statistical analysis of parasite prevalence and mosquito abundance

To examine the association of months and sampling areas with mosquito abundance and parasite prevalence in mosquitoes, data were consolidated by sampling week. As data were overdispersed and not normally distributed (Anderson-Darling normality test, p < 0.001), a generalised linear model was constructed with a quasipoisson family for abundance analysis and with a quasibinomial family for prevalence analysis. Initially, month, area and their interaction were used as explanatory variables, but as their interaction showed no relevant effects, it was removed and final models were constructed for months and areas separately. To compare the prevalence between mosquito species, a Chi-squared test of independence was carried out.

### 2.12 Sequence clustering

Each DNA sequence produced in this study was compared to the GenBank (nr) database using BLASTn; a best match to *Plasmodium* spp. confirmed that the sample was derived from an avian malaria parasite. BLAST analysis was unable to identify sequences to lineage level due to high similarity among *cytb* sequences of the lineages. To ensure the most comprehensive identification of the sequences, we combined all *Plasmodium* sequences from our sampling (n = 521) into a multiple sequence alignment with every *Plasmodium* unique *cytb* sequences stored in GenBank and previously sampled from birds (n = 2210). To obtain the reference sequences, a single *Plasmodium cytb* sequence was used as a query in a BLASTn to search up to 10,000 matches ordered by BLAST score. The results included non-avian *Plasmodium*, *Haemoproteus* and *Leucocytozoon* sequences at the lower end, ensuring that all available avian *Plasmodium* sequences were comprised. Avian *Plasmodium* sequences were selected and aligned using MEGA (Kumar et al., 2018). Identical GenBank sequences were removed to make the alignment non-redundant; sequences containing ambiguous bases were removed, as were incomplete sequences, producing a 378 bp alignment of 2731 sequences.

Our approach to classifying these sequences was to cluster with CD-HIT (Fu et al., 2012), applying a 1% sequence identity threshold, such that sequences displaying <1% divergence were considered the same lineage. This threshold was adopted for two reasons; first, it lies within the recognized margins of intraspecific sequence variation (i.e. 1-4% (Outlaw and Ricklefs, 2014); and second, at this identity level named sequences from most morphospecies were retained within single clusters.

### 2.13 Phylogenetic analysis

To estimate a phylogeny of our sequence collection, we aligned representative mosquito sequences, representative sequences from the clusters identified in the CD-HIT analysis, and all sequences derived from wild birds and penguins. The sequences were aligned using ClustalW (Hall, 2011) and *Leucocytozoon* was used as outgroup to root the tree. A maximum likelihood phylogeny was estimated using MEGA (Kumar et al., 2018) with a GTR + G model (estimated by Smart Model Selection using the Akaike Information Criterion (Lefort et al., 2017)). Node robustness was estimated using an SH-like log-Likelihood ratio test (Anisimova and Gascuel, 2006). The phylogeny included morphospecies sequence vouchers from the MalAvi database (Bensch et al., 2009), and the morphospecies name was then given to the clade into which the voucher fell.

## 3 Results

### 3.1 Avian malaria outbreak in the Humboldt penguins

The penguin colony (n = 44) was temporarily relocated to an indoors exhibit, whilst theirs was being refurbished. When the penguins were moved, blood smears (n = 13) and blood samples (n = 14) were taken from 21 penguins, all of which were negative for *Plasmodium* spp. Approximately three weeks later non-specific clinical signs were observed, such as anorexia, depression, weight loss, lethargy and regurgitation. Thereafter, signs progressed to ataxia, dyspnoea and opisthotonos, leading to death or euthanasia. Sudden deaths without clinical signs were also observed (signs and lesions are presented individually in Table 1). Penguins received anti-malarial treatment with primaquine and chloroquine, antibiotics or anti-fungal drugs depending on the suspected cause of disease. Further blood smears (n = 2) and blood samples (n = 7) were analysed during the event, with all of them being negative to *Plasmodium* spp. (Table 2). The mortality event produced 22 casualties, which were observed between September 29th^,^ 2017 and January 21st^,^ 2018, with the majority of them occurring in October and November (Table 2).

**Table 1:**
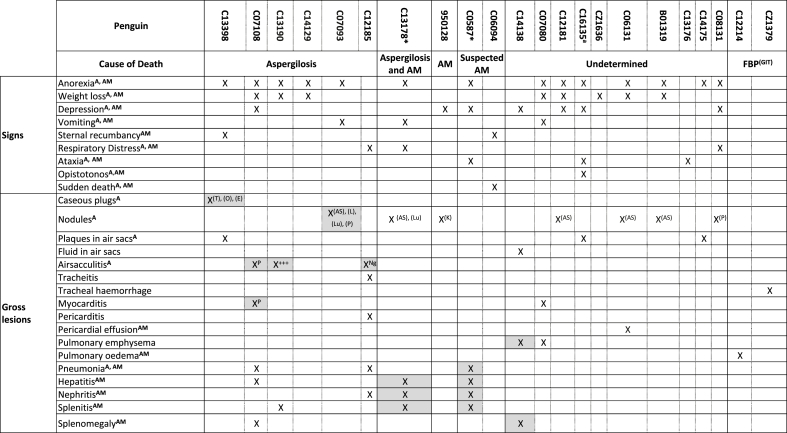

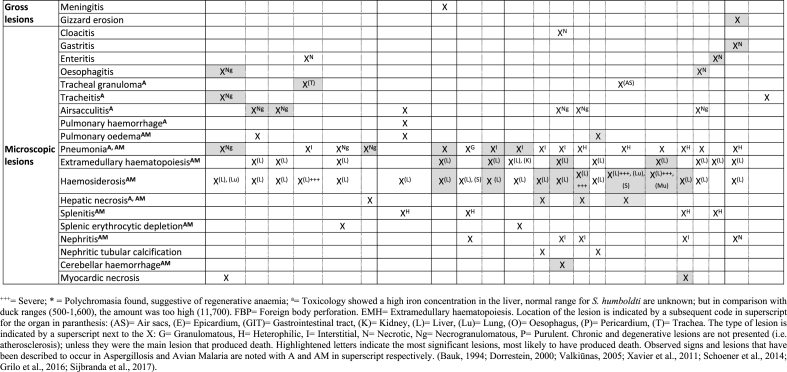
Signs and lesions presented in the 22 penguins that died during the mortality event, indicating the most significant lesions that contributed to the establishment of a cause of death.

**Table 2:** Avian malaria diagnostic tests done on penguins that died during the mortality event.

ID	Death date	Blood smear	Blood sample PCR	Organ PCR testing						CISH	COD
				Brain	Heart	Liver	Lung	Spleen	Kidney		
CO587	29/Sep/17	–	N	–	P	N	P	–	–	N	AM s
C13178	05/Oct/17	N	–	–	N	N	P	N	N	N	Aspergillosis AM s
C06094	08/0ct/17	–	–	P	N	P	P	–	–	N	AM s
C12185	10/Oct/17	N	N	N	N	N	N	N	–	N	Aspergillosis
C07093	14/Oct/17	N	–	N	N	N	–	–	–	N	Aspergillosis
950128	15/Oct/17	–	–	N	P	P	–	–	–	P	AM
C14138	15/Oct/17	N	N	N	N	N	N	N	N	N	Undetermined
C07080	17/Oct/17	–	N	N	N	N	N	N	N	N	Undetermined
C12181	23/Oct/17	N	–	N	N	N	–	N	N	N	Undetermined
C16135	24/Oct/17	–	N	N	N	N	N	N	N	N	Undetermined
CZ1636	30/Oct/17	N	N	N	N	N	–	–	N	N	Undetermined
C06131	06/Nov/17	–	N	N	N	N	N	N	N	N	Undetermined
C14129	08/Nov/17	N	N	N	–	N	N	N	N	N	Aspergillosis
CZ1379	10/Nov/17	N	N	N	N	N	N	N	N	N	Foreign body perforation of GIT
BO1319	16/Nov/17	N	N	N	–	N	–	N	N	N	Undetermined
C13190	17/Nov/17	–	–	–	–	–	–	–	–	N	Aspergillosis
C12214	17/Nov/17	–	N	P	P	P	–	P	–	N	Foreign body perforation of GIT*
C13176	21/Nov/17	–	–	N	–	–	N	N	–	N	Undetermined
C14175	14/Dec/17	–	–	–	–	–	–	–	–	N	Undetermined
C08131	02/Jan/18	N	–	–	–	–	–	–	–	N	Undetermined
C07108	13/Jan/18	–	–	–	N	N	–	–	N	N	Aspergillosis
C13398	21/Jan/18	–	–	–	–	–	–	–	–	N	Aspergillosis

ID = Identification; PCR = Polymerase chain reaction; CISH= Chromogenic in situ hybridization; COD = Cause of death; N = Negative; P = Positive; AM = Avian malaria; s = Suspected; GIT: gastrointestinal tract; *: The organ lesions caused by *Plasmodium* spp. in this penguin were mild, whereas the foreign body caused significant damage.

### 3.2 Pathological examination in dead penguins

**Fig. 2:**
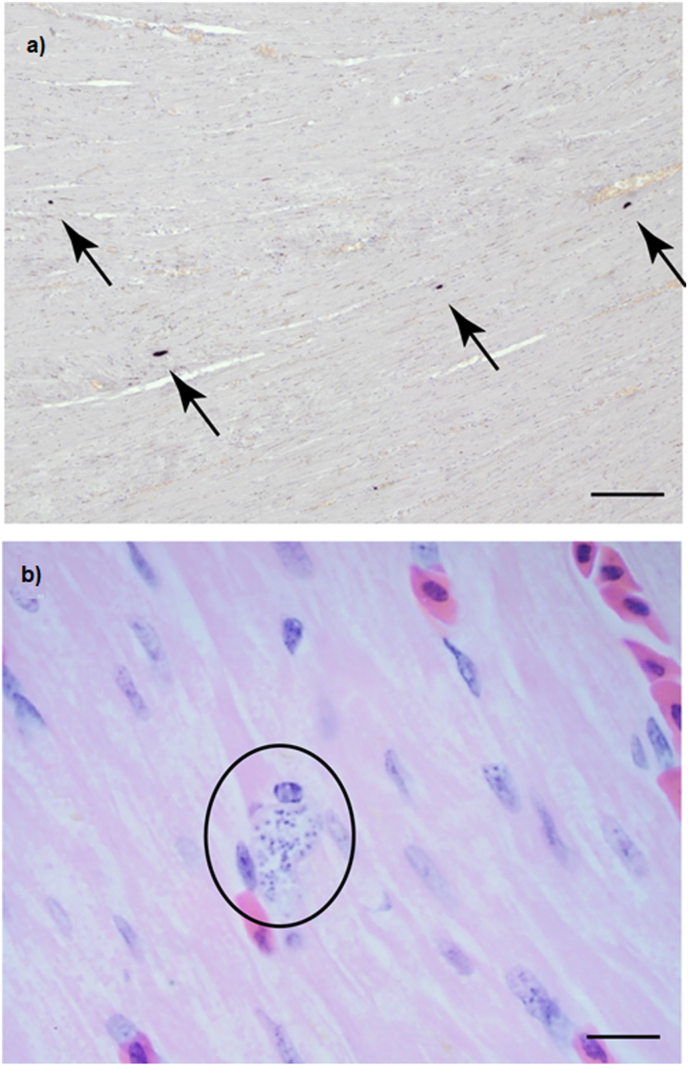
**Histopathology associated with mortality of a Humboldt penguin (*Spheniscus humboldti*) infected with *Plasmodium* sp**. Penguin paraffin-embedded heart tissue section (5 μm) stained with haematoxylin and eosin **a.** Four sites of chromogenic in situ hybridization with a *Plasmodium*-specific probe occurred in what appeared to be cardiac macrophages. Magnification: x10. **b.** Same Penguin heart tissue sections inspected under light microscopy. Magnification: x100. Exoerythrocytic meronts are seen breaking out of a cardiac macrophage (ellipse).

The 22 penguins that died were examined post-mortem. All birds displayed a non-specific diffuse vascular congestion in parenchymal organs, with enlargement and rounded edges of the liver, spleen and kidney. Five of 22 animals were positive by PCR for *Plasmodium* (Table 2); these birds displayed histopathological changes in the form of mild multifocal hepatic portal extramedullary haematopoiesis and haemosiderosis. Four of twenty-two birds showed a mild portal necrotising hepatitis, and these cases often showed a mild heterophilic splenitis and interstitial pneumonia (Table 1). Low numbers of exoerythrocytic meronts were identified in the heart and pulmonary endothelium by histopathology in one penguin. That same penguin showed the positive results of CISH with a *Plasmodium*-specific probe to heart tissue sections (Fig. 2a) and identified emerging meronts at higher magnification (Fig. 2b) (Table 2).

Established causes of death (COD) during the mortality event were aspergillosis (n = 7), avian malaria (n = 1), suspected avian malaria (n = 3) and foreign body perforation of the gastrointestinal tract (n = 2). For the remaining cases (n = 10), a COD could not be ascertained, but a multisystemic infection was evident by the lesions observed; amongst the many possible etiological agents for those lesions *Aspergillus* spp. and *Plasmodium* spp. are included (Table 1). Opportunist bacterial pathogens were also isolated (4/22), such as *Providencia rettgeri* (bone marrow and air sacs), *Aeromonas caviae* (lung and air sacs) and *Citrobacter freundii* (air sacs).

### 3.3 Avian malaria screening in wild birds

In total, 81 free-wild birds belonging to seven orders, 17 families and 27 species were recovered from Chester Zoo grounds during the study period. From these, only two Eurasian blackbirds (*Turdus merula*) were positive for *Plasmodium* spp. (See Table A in S1 File)

### 3.4 Mosquito abundance at Chester Zoo during 2017

**Fig. 3:**
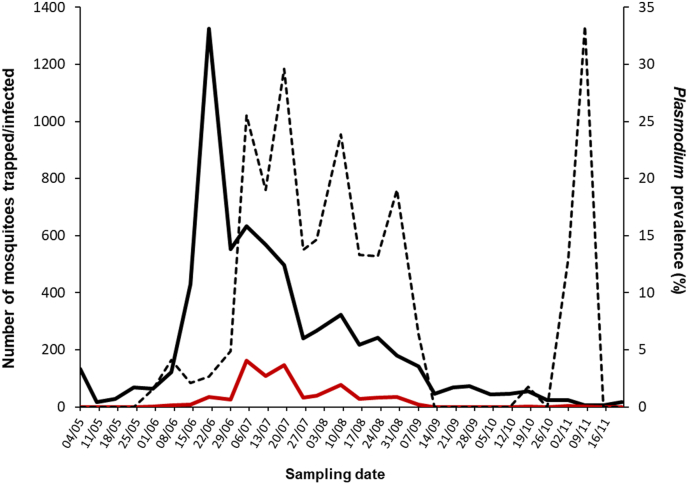
**Temporal distribution of mosquito and *Plasmodium* spp. prevalence at Chester Zoo between May and November 2017**. Continuous top line: total number of mosquitoes collected on a weekly basis; Continuous bottom line: total number of *Plasmodium* infections; Dashed line: parasite prevalence estimated as a proportion of infected mosquitoes of the total captured on a weekly basis.

Between May and November 2017, 6862 female mosquitoes belonging to seven species were collected across the ten sampling sites at Chester Zoo. Of these, *Culex pipiens* (n = 5345) was the dominant species (77.9%), followed by *Culiseta annulata* (n = 238; 3.5%). Minor contributions were made by *Anopheles claviger* (n = 2)*, An. maculipennis* s.l. (n = 5)*, An. plumbeus* (n = 1)*, Cx. torrentium* (n = 48), and *Culiseta morsitans* (n = 3). A minority of specimens were damaged and only partially identified: *Aedes* spp. (n = 1), *Anopheles* spp. (n = 3), *Culex* spp. (n = 818), *Culiseta* spp. (n = 20) and Culicinae (n = 378). Significant difference on mosquito abundance were observed among collection months or sampling sites. More mosquitoes were caught from June to August than during other months, and the lowest numbers were observed in May and November (Fig. 3) (Table B in S1 File), while significantly higher numbers of mosquitoes were trapped at sites A1 (P < 0.001) and A3 (P = 0.008) (Fig. 1) (Table C in S1 File).

### 3.5 Plasmodium prevalence in mosquitoes

In total, 753 mosquitoes tested positive by PCR out of 6459 analysed (11.7%). The number of mosquitoes infected with *Plasmodium* spp. increased as the year progressed from zero at the beginning of May to a peak of 147/496 (29.6%) mosquitoes trapped during mid-July (Fig. 3). Parasite prevalence in mosquitoes remained high into early August (77/323; 23.8%) as mosquito abundance gradually fell, before declining sharply to 6.3% (9/142) in early September. Infected mosquitoes were then not observed, apart from a minor recrudescence in November when 3/23 (13%) and 2/6 (33.3%) mosquitoes were PCR-positive.

From the seven species of mosquitoes identified at Chester Zoo during 2017, *Plasmodium* DNA was only detected in *Culex pipiens* and *Culiseta annulata*. In total, 11.4% (619) of *Cx. pipiens* and 10.3% (25) of *Cs. annulata* mosquitoes were positive respectively; a difference in prevalence that is non-significant (χ^2^ = 0.18, df = 1, P = 0.67). There were also PCR mosquito positives that could not be fully identified, including 96 *Culex* spp., one *Culiseta* spp. and 37 as Culicinae.

Parasite prevalence was higher in July and August, but not significantly different (Table D in S1 File). There was a difference in *Plasmodium* prevalence by trapping site, with A2 having a significantly higher prevalence (18%) than the other areas (Table E in S1 File) (Fig. 1).

### *3.6* *Cytochrome b* (*cytb*) *sequence clustering and phylogenetic analysis*

The data set contained 496 *cytb* sequences derived from mosquitoes trapped at Chester Zoo, two sequences obtained from wild birds as well as 23 penguin-derived sequences from Chester Zoo (n = 8), London Zoo (n = 2), Paignton Zoo (n = 9), Blackpool Zoo (n = 3) and Cotswold Wildlife Park (n = 1). Penguin sequences are listed in Table 3 and all sequences produced in this study are catalogued in Table F (see S1 List).

**Table 3:** Twenty-three *Plasmodium cytb* sequences obtained in this study from penguins (*Sphensicus* spp., *Eudyptes chrysolophus*) known to have been infected with avian malaria in five UK zoological collections. Reference lineages found in the same cluster as our sequences in the cluster analysis at 1% divergence are given; other sequences were not previously observed. Asterisks (*) denote sequences that were collected from Chester Zoo during the 2017 mortality event.

Genbank accession	Species	Lineage	Host	Collection	Year
MW813993	*P. matutinum*	LINN1	*Spheniscus humboldti*	Chester Zoo	2017*
MW813994	*P. matutinum*		*Spheniscus humboldti*	Chester Zoo	2017*
MW814009	*P. matutinum*	LINN1	*Spheniscus humboldti*	Chester Zoo	2017*
MW814010	*P. matutinum*	LINN1	*Spheniscus humboldti*	Chester Zoo	2017*
MW814011	*P. matutinum*	LINN1	*Spheniscus humboldti*	Chester Zoo	2017*
MW813997	*P. matutinum*	LINN1	*Spheniscus humboldti*	Chester Zoo	2013
MW813999	*P. relictum*	GRW11	*Spheniscus humboldti*	Chester Zoo	2018
OM912815	*P.matutinum*	LINN1	*Spheniscus humboldti*	Chester Zoo	2018
MW813995	*P. matutinum*	LINN1	*Spheniscus magellanicus*	Blackpool Zoo	2017
MW814012	*P. relictum*	GRW11	*Spheniscus magellanicus*	Blackpool Zoo	2017
MW813996	*P. relictum*	SGS1	*Spheniscus magellanicus*	Blackpool Zoo	2018
MW813998	*P. vaughani*	AH0824	*Spheniscus humboldti*	Cotswold Wild Animal Park	2017
MW814013	*P. relictum*	SGS1	*Spheniscus humboldti*	London Zoo	2017
MW814014	*P. matutinum*	LINN1	*Spheniscus humboldti*	London Zoo	2017
MW814001	*P. matutinum*	LINN1	*Eudyptes chrysolophus*	Paignton Zoo	2013
MW814003	*P. relictum*		*Eudyptes chrysolophus*	Paignton Zoo	2013
MW814005	*P. matutinum*		*Eudyptes chrysolophus*	Paignton Zoo	2013
MW814000	*P. matutinum*	LINN1	*Spheniscus humboldti*	Paignton Zoo	2017
MW814002	*P. matutinum*	LINN1	*Spheniscus humboldti*	Paignton Zoo	2017
MW814004	*P. matutinum*	LINN1	*Spheniscus humboldti*	Paignton Zoo	2017
MW814006	*P. relictum*	GRW11	*Spheniscus humboldti*	Paignton Zoo	2017
MW814007	*P. matutinum*	LINN1	*Spheniscus humboldti*	Paignton Zoo	2017
MW814008	*P. matutinum*	LINN1	*Spheniscus humboldti*	Paignton Zoo	2017

From all the sequences produced in this study, 442 clustered in four different groups, each containing a single reference sequence from GenBank with an associated lineage (Table 4). One cluster corresponds to the LINN1 lineage and contains 288 sequences, including 272 mosquito-derived sequences, six Chester Zoo penguin sequences from 2017 (n = 4), 2013 (n = 1) and 2018 (n = 1), two wild birds (Eurasian blackbirds), and eight penguins from Paignton Zoo (n = 6), Blackpool Zoo (n = 1) and London Zoo (n = 1). A second cluster corresponds to the SYAT05 lineage, containing 126 mosquito-derived sequences and one penguin-derived sequence from the Cotswold Wildlife Park collection. A third cluster corresponds to the GRW11 lineage, which contains 17 sequences including 13 mosquito-derived sequences, one penguin-derived sequence from Chester Zélé et al. (2014), and three from Paignton Zoo (n = 1), Blackpool Zoo (n = 1) and London Zoo (n = 1). The last cluster is that of the SGS1 lineage, comprising nine mosquito-derived sequences and one penguin sequence from Blackpool Zoo. From the remaining 79 mosquito-derived sequences, 72 formed their own cluster, each of which may represent a new lineage, and 7 clustered together without a reference sequence suggesting they are one new lineage. Given that we cannot entirely rule out sequencing error, we took a conservative approach to interpreting the number of distinct lineages among mosquito-derived sequences, counting only those that were observed in at least two individual mosquitoes (Table 4).

**Table 4:** *Cytb* lineages observed in three *Plasmodium* species infecting mosquitoes and penguins. (^a^) denote lineages identical to established strains, other sequences were not previously observed. Asterisks (*) denote lineages that were collected from Chester Zoo penguins during the 2017 mortality event.

Species	*P. matutinum*	*P. vaughani*	*P. relictum*	*P. relictum*	*P. matutinum*	*P. matutinum*	*P. vaughani*	*P. relictum*
Lineage	LINN1^a^ (n)	SYAT05^a^ (n)	SGS1^a^ (n)	GRW11^a^ (n)	New (n)	Unique^a^ (n)	Unique^a^ (n)	Unique^a^ (n)
Chester Zoo mosquitoes 2017	272	126	9	13	7	48	18	3
Chester Zoo wild birds 2017	2							
Chester Zoo penguins 2017	4*					1*		
Chester Zoo penguins 2013	1							
Chester Zoo penguins 2018	1			1				
Paignton Zoo penguins	6			1		1		1
Blackpool Zoo penguins	1		1	1				
London Zoo penguins	1			1				
Cotswold Wildlife Park penguins		1						
Total	288	127	10	17	7	50	18	4

a All sequences grouped here are different amongst themselves.

All of the lineages that clustered with our sequences belong to known parasite morphospecies. The lineage LINN1 belongs to *P. matutinum;* this group contained 4/5 *Plasmodium* sequences recovered from penguins that died at Chester Zoo in 2017. The lineage SYAT05, corresponds to the species *P. vaughani*, which includes one case from a Humboldt penguin from Costwold Wildlife Park (GeneBank accession number MW813998), and is the first *P. vaughani* infection reported in penguins. The lineages GRW11 and SGS1 both derive from *P. relictum*, and neither of them were found in any of the dead penguins during the 2017 outbreak; although *P. relictum* was detected in 2018 in a penguin that survived infection (GeneBank accession number MW813999).

**Fig. 4:**
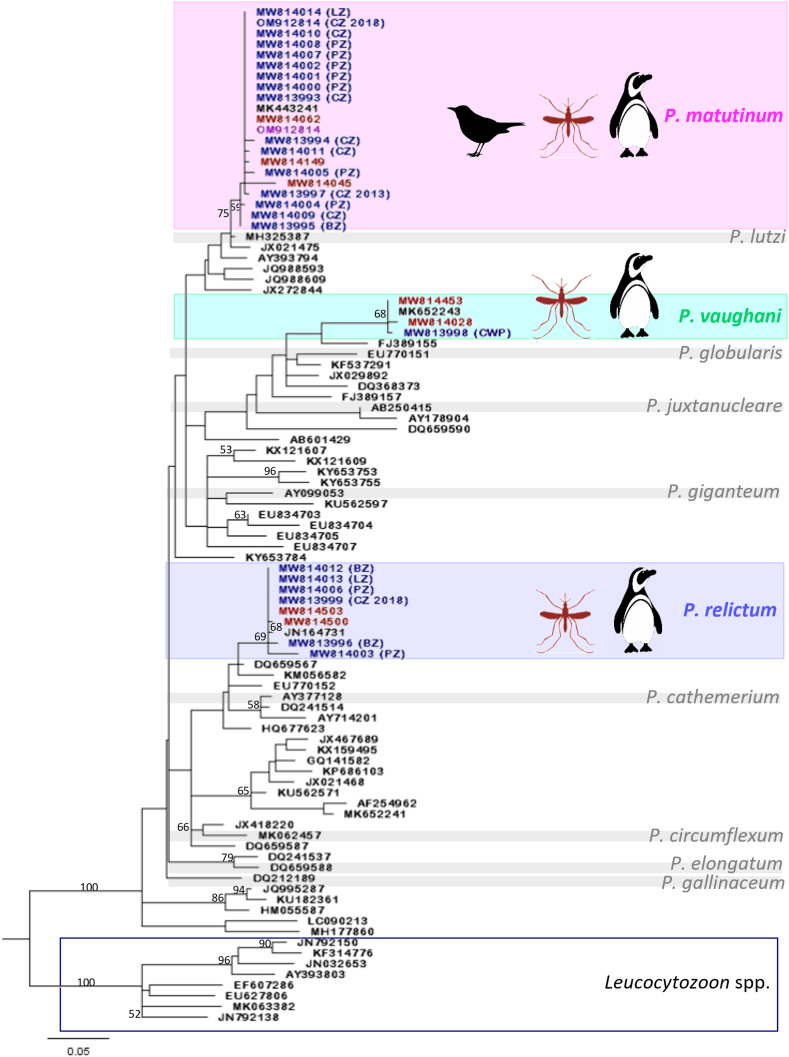
**Maximum likelihood phylogeny of *Plasmodium* spp. *cytb* sequences**. The phylogeny was estimated from a 378bp multiple sequence alignment using a GTR+Γ+Ι model (α = 0.488; proportion of invariant sites = 0.248). The tree is rooted with an outgroup of *Leucocytozoon* sequences (boxed). Node accuracy is indicated by an SH-like log-Likelihood ratio metric; bootstrap values greater than 0.5 are displayed in the tree. Novel sequences obtained in this study are shaded with their corresponding reference sequence; *P. matutinum* (MK443241), *P. vaughani* (MK652243) and *P. relictum* (JN164731). The clusters contain sequences derived from penguins, mosquitoes or wild birds, which is indicated by a penguin, mosquito or a bird symbol. One wild bird sequence is present in the *P. matutinum* cluster (OM912814); and three (MW814062, MW814149, MW814045), two (MW814453, MW814028) and two (MW814503, MW814500) mosquito sequences are present in the *P. matutinum*, *P vaughani* and *P. relictum* clusters respectively. The rest of the sequences in those clusters correspond to 23 novel sequences from penguins infected in the UK, indicating their origin (CZ: Chester Zoo, LZ: London Zoo, PZ: Paignton Zoo, BZ: Blackpool Zoo, CWP: Cotswold Wildlife Park) and year of sampling, if not 2017. Other shade sequences correspond to recognized morphospecies. *P. vaughani* cluster represents 145 novel sequences, *P. matutinum* cluster represents 345 novel sequences and *P. relictum* clusters represents 31 novel sequences. (For interpretation of the references to colour in this figure legend, the reader is referred to the Web version of this article.)

A maximum likelihood *cytb* phylogeny displaying the relationships among mosquito- and penguin-derived sequences is shown in Fig. 4. Nodes' robustness was assessed with an SH-like log-Likelihood ratio metric (Anisimova and Gascuel, 2006); non-parametric bootstrapping was carried out, but the tree topology was generally not supported by bootstrap values > 50 due to the relatively small number of characters available. Nevertheless, the topology resolves established morphospecies and largely agrees with previous estimates of their phylogenetic relationships (Valkiūnas et al., 2007; Valkiūnas and Iezhova, 2017), which provides some external validation of the topology. The tree shows how mosquito, wild-bird and all penguin-derived *Plasmodium* sequences obtained from Chester Zoo and another four UK locations adopt one of three positions within the topology, each well supported by a log-Likelihood ratio test. These clusters include Malavi sequence vouchers for *P. relictum*, *P. matutinum* and *P. vaughani*, confirming the clustering result. Of the species circulating in vectors at Chester Zoo in 2017, only *P. matutinum* was observed in infected dead penguins during the mortality event.

## 4 Discussion

This study evaluated the distribution of *Plasmodium* among mosquitoes and birds during a seven-month survey at Chester Zoo, carried out before and during a Humboldt penguin mortality event. Parasite species found in mosquitoes belonged to three species; *P. matutinum*, *P. vaughani* and *P*. *relictum*. Eurasian blackbirds were the only free-living wild bird species infected and the species identified was *P*. *matutinum*. Likewise, the Humboldt penguins were found infected with *P. matutinum* (5/22)*,* four of them with the same lineage LINN1. This is, to our knowledge, the first report of *P. matutinum* causing an avian malaria outbreak within a mortality event. *P. matutinum*, *P. relictum* and *P*. *vaughani* were also identified from penguin samples of other UK zoo collections. Noticeably, no previous reports of *P. vaughani* producing casualties in penguins were found.

An overall *Plasmodium* prevalence within the mosquito population of 11.7% was observed, with a peak during July and August, which follows the peak in mosquito abundance in June. This is consistent with previous observations of similar studies (Zélé et al., 2014) and corresponds with the peak of avian malaria outbreaks reported before in the UK (Hernandez-Colina et al., 2021a). The high prevalence in November samples is belied by the very small numbers of trapped mosquitoes and this observation does not contradict a mid-summer peak. Abundance was highest in sampling areas which are close to favourable breeding sites (i.e. ponds with shallow edges and rich in organic matter), like A1 and A3 (Fig. 1), and thus mosquitoes could have been caught after emerging as adults and before dispersing. By contrast, the area with significantly higher prevalence (A2, 18%) did not yield as many mosquitoes as others; this area is adjacent to the penguins' exhibit, which could represent a higher risk of infection for the penguins. These differences must be considered for future mosquito surveillance and control activities. *Cytb* sequences from 496 positive mosquitoes belonged to three *Plasmodium* species: *P. matutinum* (327, 65.9%), *P. vaughani* (144, 29%) and *P. relictum* (25, 5.1%), all of which are common parasites in birds and mosquitoes across Europe and beyond (Spottiswoode et al., 2020).

*P. matutinum* was responsible for all malaria infections in penguins observed during the mortality event, and four of the five infected penguins had the lineage LINN1; this is the first time that *P. matutinum* and its lineage LINN1 have been associated with an avian malaria outbreak. *Culex pipiens* was the most abundant mosquito species recorded on site, it has been found feeding on penguins at the zoo (Hernandez-Colina et al., 2021b); hence, it is most likely responsible for *Plasmodium* transmission to penguins as it was demonstrated to be a vector; however, identifying the wild bird reservoir requires further investigation. Additionally, Blackbirds were the only wild bird species identified infected with *P. matutinum* in this incident.

During the mortality event at Chester Zoo, *Plasmodium* infection was not observed in penguin blood smears or detected in blood samples by PCR; but it was confirmed by organ PCR in five of the 22 penguins that died. Of these, avian malaria was confirmed by histopathological and CISH evidence in only one individual. Such difficulty in detecting avian malaria infections either in live or dead individuals has long been observed particularly in the case of penguins (Igunas et al., 2016). For instance, Fix et al. (1988) described difficulties in diagnosing avian malaria from blood smears, detecting parasitaemia in only one of 46 infected penguins. Grilo et al. (2016) have pointed out that acute infections can cause mortality without parasitaemia occurring, making the detection of the infection impossible from blood samples. Furthermore, in this case, detected infections were produced by *P. matutinum*, a species known to produce low parasitaemia (Spottiswoode et al., 2020). This species has been associated to Humboldt penguin mortality in only one previous occasion (Valkiūnas et al., 2017); hence, they may not be a competent host for *P. matutinum*, resulting in an abortive infection in which gametocytes do not develop, and so cannot be diagnosed by blood analysis (Palinauskas et al., 2016; Valkiūnas and Iezhova, 2017). Abortive infections in penguins are not uncommon, and that produced by *P. elongatum* is a well-known and documented case (Palinauskas et al., 2016).

Diagnosis of avian malaria from organs has also represented a challenge. Fix et al. (1988) confirmed avian malaria by histopathology in 13 out of 20 penguins that presented characteristic lesions of avian malaria, all of which have been receiving antimalarial treatment. Spottiswoode et al. (2020) studied penguins under malaria prophylaxis; nine penguins with high clinical suspicion of death by avian malaria were tested, where all organ smear impressions were negative and five tested positive by PCR. The hypothesis of anti-malarial treatment interfering with diagnostic methods is supported by the observation of degenerated parasites in blood smears of penguins treated with chloroquine (Vanstreels et al., 2019). Likewise, detection of the parasite in suspected cases seems to be more effective when treatment is not applied; for instance, Augusto-Taunde et al. (2019) positively detected *Plasmodium* parasites by PCR in nine of ten tested samples, and Vanstreels et al. (2014) diagnosed avian malaria by PCR and histopathology in three dead penguins suspected to die from avian malaria. Another possibility for the absence of positive results in highly suspicious cases might be associated to an abortive infection where the parasite's development is incomplete, or to the effect of anti-malarial drugs administration, where the treatment may have cleared most of the parasites from the tissues at a late stage despite irreversible damage having already occurred to the organs. It should also be considered that in some cases, the lack of experience of veterinarians and pathologists may prevent the accurate and prompt diagnosis of the disease; however, this expertise is mostly relevant for parasite species identification (Valkiūnas, 2005; Grilo et al., 2016).

Avian malaria was confirmed as the COD for one penguin, and it was highly suspected to be responsible for another three deaths; as no cases were reported before, this can be considered as an outbreak (WOAH, 2022). An outbreak investigation typically looks to establish a place and time for the observed cases (CDC, 2022), and in this event, these criteria were clearly established as the penguin colony was housed in the same facility and deaths occurred during a relatively short period. Another critical aspect of an outbreak investigation is the setting of a case definition (Kane and Morley, 1999; CDC, 2022), which at present is not standardised for avian malaria. The investigation of this event did not defined avian malaria cases a priori, instead it was based on establishing CODs and classifying cases as confirmed or suspected. Cases were considered as confirmed if *Plasmodium* spp. was observed by histopathology or CISH in lesions that produced death of the individuals and that are typically associated to the disease. Suspected cases were those in which the death was attributed to lesions associated to avian malaria and with PCR detection of the parasite. Early stages of outbreak investigation commonly employ diverse case definitions to allow for diagnosis uncertainty (e.g., suspected, possible, probable, confirmed) based on clinical criteria, time, place, and presence or absence of diagnostic tests (CDC, 2022). Application of these criteria a priori might be particularly useful in the diagnosis of avian malaria, since the disease is challenging to diagnose as discussed previously, and most of its signs and lesions are unspecific or shared with other diseases.

The other infectious COD observed here was aspergillosis, which should be considered as a main differential aetiology in the investigation of avian malaria outbreaks, since both pathogens are not mutually exclusive and have been reported simultaneously in penguin mortalities (Fix et al., 1988; Vanstreels et al., 2015a; Grilo et al., 2016). Moreover, we confirmed a mixed infection in one individual (C13178). Likewise, other viral infections and toxins should be considered as differential diagnosis, since they require specific tests and could be missed from routine examinations. During the analysis of the event, other CODs were found; however, for a large number of penguins which presented lesions indicative of a multisystemic infection, a COD could not be established despite great diagnostic effort involving histopathology, bacterial and fungal cultures and *Plasmodium* PCR testing. Many of these cases presented one or more signs and lesions consistent, but not exclusive, of avian malaria or aspergillosis, and given the event frame, there is a possibility that they were caused by the same pathogens.

In summary, *Plasmodium* spp. and *Aspergillus* spp. could have played a more important role in the mortality event either as the main pathological agents or as secondary infections beyond what was possible to prove. Hence, there is a need for epidemiological definitions of cases for both agents that will aid to develop a systematic investigation during mortality events and help to clarify the roles of these microorganisms in future outbreaks.

The genetic analyses integrate 521 new *cytb* sequences produced here with all avian *Plasmodium* sequences seen before. The new sequences obtained belong to one of three species, since they adopted one of three positions within the tree, corresponding with the voucher sequences for *P. matutinum, P. vaughani* and *P. relictum*. Lineage definition is a persistent challenge in systematic studies of avian malaria (Outlaw and Ricklefs, 2014); currently, there are multiple criteria to establish lineages, all based upon sequence variation of a conventional molecular marker, the *cytb* gene (Martinsen et al., 2007). Prior to phylogenetic reconstruction, sequence clustering was used to remove redundancy among sequences, applying a 1% sequence divergence value as the threshold for cluster inclusion. This value is well within the accepted range of empirical values seen in accepted haemosporidian species (Outlaw and Ricklefs, 2014). It is a conservative measure that maintains the integrity of well described morphospecies, ensuring that all sequences ascribed to each species cluster together, and at the same time preventing the creation of new lineages based on a single base change that could be attributed to sequencing or editing errors.

While three *Plasmodium* species were circulating at Chester Zoo in 2017, only *P. matutinum* was seen infecting penguins. *P. matutinum* infection has been recorded in diverse birds in North America, Europe, Asia and New Zealand (Sijbranda et al., 2017). Besides the infected penguins at Chester Zoo in 2017, 2018 and 2013, *P. matutinum* was also identified in penguins from Paignton Zoo, Blackpool Zoo and London Zoo. Clearly, *P. matutinum* is a cosmopolitan parasite that circulates within penguin collections across the UK and could be associated with more penguin deaths than what was proven here.

Since *P. vaughani* and *P. relictum* were present in mosquitoes but not in penguins at Chester Zoo during this survey, our results may reflect temporal variation in parasite species pathogenicity of the kind suggested by Spottiswoode et al. (2020). They observed that, over 20 years, the parasite species obtained from avian malaria fatalities (i.e. *P. cathemerium* SEIAUR01, *P. matutinum* LINN1) were different to those found in the blood of live birds sampled in the final three years of the study (i.e. *P. relictum* SGS1). Then again, a *P. vaughani* infection was observed in a penguin at the Cotswold Wildlife Park in 2017, and *P. relictum* infections at both Paignton Zoo and Blackpool Zoo over several years (see Table 3). Indeed, *P. relictum* is another highly cosmopolitan parasite that is frequently associated with avian malaria in penguins (Lapointe et al., 2012; Schoener et al., 2014; Palinauskas et al., 2008). Thus, it is likely that both *P. vaughani* and *P. relictum* may contribute to avian malaria at Chester Zoo, although this was not recorded in the penguins. In fact, testing for avian malaria continued at Chester Zoo in 2018 and a penguin was found to be infected with *P. relictum* (lineage GRW11; MW813999), surviving on this occasion.

Besides species variation, five *cytb* lineages, (based on a 1% divergence with sequences observed at least twice), were seen in *P. matutinum* (n = 2), *P. vaughani* (n = 1) and *P. relictum* (n = 2). Not all strains of a given *Plasmodium* species appear to be equally pathogenic; for example, Valkiūnas et al. (2017) commented that while an Italian strain of *P. matutinum* was lethal, an American strain was much less virulent (Valkiūnas and Iezhova, 2017). Similarly, two *P. matutinum* lineages over the period when penguin malaria cases occurred were observed, but 4/5 fatalities were associated with the lineage LINN1. This could be a simple sampling effect, given that this lineage was the most common one (61% of all *P. matutinum* sequences), and so, had the greatest chance of infecting the penguins. Yet, it might also indicate that LINN1 has a higher intrinsic reproduction rate and is more virulent than other strains in Humboldt penguins.

Differences in virulence have also been observed for the highly cosmopolitan species *P. relictum,* the model avian *Plasmodium*. Beadell et al. (2006) recognized that what is consider as *P. relictum* is a complex of genetic lineages that form a robust clade in phylogenies, this genetic variation among lineages affects virulence (Vanstreels et al., 2015b). This parasite is recognized as one of the most pathogenic species (Grilo et al., 2016), yet there is evidence that certain lineages benefit host reproductive output (Lachish et al., 2011), and in this study a *P. relictum* infection was observed in 2018 in a penguin that survived infection. *P. relictum* is a notable generalist, recovered from 127 different host species from 11 orders (Vanstreels et al., 2015b; Fecchio et al., 2020), and it may be that virulent *P. relictum* lineages result from maladaptation following the infection of non-competent hosts, which produce abortive infections with high mortality.

*P. matutinum* was associated with mortality in Humboldt penguins and was observed circulating widely in mosquitoes before and during the mortality event at Chester Zoo in 2017. The overall prevalence on mosquitoes was 11.7% with a peak in July and August, when the highest transmission risk could be expected. Infections in penguins were only associated with one of three detected *Plasmodium* spp. species (*P. matutinum*), and only two lineages (LINN1 and New), suggesting that not all *Plasmodium* parasites present the same threat to penguins. *P. matutinum* LINN1 was also associated with penguin fatalities at other British zoos in 2013 and 2017, which may indicate a particular risk to these birds in the UK. The association of particular genotypes with virulence must be confirmed statistically and clinically; thereafter, finding these during surveillance could be a trigger for initiating specific actions to prevent fatalities. Host associated factors such as immune system competence, concomitant infections, and nutritional and physiological state should also be considered in relation to the susceptibility and seriousness of infection, as shown in this multicausal event. More long-term surveillance is required to achieve a sound scientific basis for management advice to establish a statistical association between *Plasmodium* infection and mortality in the frequent absence of definitive diagnosis, and to determine if specific lineages pose threats to the penguin collection at Chester Zoo and elsewhere.

## Ethics approval and consent to participate

Ethical approval for the work carried out in this study was obtained from the Chester Zoo science committee and University of Liverpool Veterinary Research Ethics Committee (reference VREC532a).

## Funding

This study was supported by PhD studentships from the Mexican Government (Conacyt) (#270724/440743) for MGO and AHC, a research grant from 10.13039/501100005359Chester Zoo and a 10.13039/100012065Houghton Trust (Avian Pathology) research award for MGO and AHC.

## Authors' contributions

*Conceptualization:* MGO, AHC, JC, MB, APJ*. Investigation:* MGO, AHC, TH, JC*. Formal analysis:* MGO*,* AHC, JC, APJ*.* Funding *acquisition:* AHC, APJ, MGO, MB*. Resources:* LE, JL*. Writing:* MGO, AHC,APJ.

## Declaration of competing interest

None.
