# Supplementary material for: Molecular and epidemiological surveillance of Plasmodium spp. during a mortality event affecting Humboldt penguins (Spheniscus humboldti) at a zoo in the UK
Source: Int J Parasitol Parasites Wildl. 2022 Jul 5;19:26–37. doi: 10.1016/j.ijppaw.2022.06.010 (PMC9403903; doi:10.1016/j.ijppaw.2022.06.010)
Supplement: Multimedia component 1 [file mmc1.docx]

**Table A.** *Plasmodium* spp. prevalence in dead free-living wild birds found in Chester zoo during 2017

| **Order** | **Family** | **Species** | **Common name** | **P (n)** | **n** | **%** |
| --- | --- | --- | --- | --- | --- | --- |
| Accipitriformes | Accipitridae | *Buteo buteo* | Eurasian buzzard |  | 2 | 0 |
| Anserifirme | Anatidae | *Anas platyrhynchos* | Mallard |  | 9 | 0 |
| Charadiformes | Laridae | Larus argentatus | European herring gull |  | 2 | 0 |
|  |  | *Larus ridibundus* | Black-headed gull |  | 5 | 0 |
| Columbiformes | Columbidae | *Columba livia* | Rock dove |  | 5 | 0 |
|  |  | *Columba palumbus* | Common woodpigeon |  | 1 | 0 |
|  |  | *Streptopelia decaocto* | Eurasian collared-dove |  | 5 | 0 |
| Gruiformes | Rallidae | *Gallinula chloropus* | Common moorhen |  | 10 | 0 |
| Passeriformes | Corvidae | *Corvus corone* | Carrion crow |  | 1 | 0 |
|  |  | *Corvus monedula* | Eurasian jackdaw |  | 1 | 0 |
|  |  | *Pica pica* | Eurasian magpie |  | 1 | 0 |
|  | Fringilidae | *Chloris chloris* | European greenfinch |  | 1 | 0 |
|  |  | *Pyrrhula pyrrhula* | Eurasian bullfinch |  | 1 | 0 |
|  | Hirundinidae | *Hirundo rustica* | Barn swallow |  | 1 | 0 |
|  | Muscicapidae | *Erithacus rubecula* | European robin |  | 1 | 0 |
|  | Paridae | *Periparus ater* | Coal tit |  | 1 | 0 |
|  |  | *Parus major* | Great tit |  | 1 | 0 |
|  |  | *Cyanistes caeruleus* | Eurasian blue tit |  | 1 | 0 |
|  | Passeridae | *Passer domesticus* | House sparrow |  | 2 | 0 |
|  | Phylloscopidae | *Phylloscopus trochilus* | Willow warbler |  | 3 | 0 |
|  | Regulidae | *Regulus regulus* | Goldcrest |  | 2 | 0 |
|  |  | *Regulus ignicapilla* | Common firecrest |  | 3 | 0 |
|  | Sturnidae | *Sturnus vulgaris* | Common starling |  | 9 | 0 |
|  | Troglodytidae | *Troglodytes troglodytes* | Northern wren |  | 1 | 0 |
|  | Turdidae | *Turdus merula* | Eurasian blackbird | 2 | 10 | 20 |
|  |  | *Turdus philomelos* | Song thrush |  | 1 | 0 |
| Pelecaniformes | Ardeidae | *Ardea cinerea* | Grey heron |  | 1 | 0 |
|  |  | Total |  | 2 | 81 | 2.5 |
|  |  |  |  |  |  |  |

**Table B.** Parameters of the GLM for the analysis mosquito abundance in relation to sampling months.

| **Variable** | **Estimate** | **Std. Error** | **t value** | **Pr(>\|t\|)** |
| --- | --- | --- | --- | --- |
| Intercept | 0.095 | 0.812 | 0.117 | 0.907 |
| October | 1.352 | 0.911 | 1.484 | 0.139 |
| September | 2.009 | 0.865 | 2.323 | 0.021 |
| August | 3.102 | 0.830 | 3.737 | <0.001 |
| July | 3.859 | 0.819 | 4.712 | <0.001 |
| June | 4.011 | 0.819 | 4.895 | <0.001 |
| May | 1.736 | 0.867 | 2.001 | 0.046 |
| Dispersion parameter for quasipoisson family taken to be 29.014.  Null deviance: 13579.3 on 299 degrees of freedom.  Residual deviance: 6744.2 on 293 degrees of freedom. | | | | |

**Table C.** Parameters of the GLM for the analysis mosquito abundance in relation to sampling areas.

| **Variable** | **Estimate** | **Std. Error** | **t value** | **Pr(>\|t\|)** |
| --- | --- | --- | --- | --- |
| Intercept | 2.552 | 0.380 | 6.708 | <0.001 |
| Area A11 | 0.579 | 0.475 | 1.218 | 0.224 |
| Area A10 | 0.364 | 0.495 | 0.735 | 0.463 |
| Area A7 | 0.199 | 0.513 | 0.389 | 0.698 |
| Area A6 | 0.085 | 0.527 | 0.161 | 0.873 |
| Area A5 | -0.133 | 0.557 | -0.239 | 0.811 |
| Area A4 | 0.492 | 0.483 | 1.020 | 0.309 |
| Area A3 | 1.169 | 0.436 | 2.683 | 0.008 |
| Area A2 | 0.068 | 0.529 | 0.128 | 0.898 |
| Area A1 | 1.506 | 0.421 | 3.581 | <0.001 |
| Dispersion parameter for quasipoisson family taken to be 55.733.  Null deviance: 13579 on 299 degrees of freedom.  Residual deviance: 11363 on 290 degrees of freedom. | | | | |

**Table D.** Parameters of the GLM for the analysis of *Plasmodium* spp. prevalence in relation to sampling months.

| **Variable** | **Estimate** | **Std. Error** | **t value** | **P (>\|t\|)** |
| --- | --- | --- | --- | --- |
| Intercept | -2.054 | 0.696 | -2.949 | 0.003 |
| October | -3.076 | 1.627 | -1.890 | 0.06 |
| September | -1.514 | 0.855 | -1.771 | 0.078 |
| August | 0.508 | 0.707 | 0.719 | 0.473 |
| July | 0.603 | 0.700 | 0.862 | 0.39 |
| June | -1.406 | 0.718 | -1.959 | 0.051 |
| May | -3.686 | 1.625 | -2.268 | 0.024 |
| Dispersion parameter for quasibinomial family taken to be 2.15.  Null deviance: 931.18 on 269 degrees of freedom.  Residual deviance: 410.93 on 263 degrees of freedom. | | | | |

**Table E.** Parameters of the GLM for the analysis of *Plasmodium* spp. prevalence in relation to sampling areas.

| **Variable** | **Estimate** | **Std. Error** | **t value** | **P (>\|t\|)** |
| --- | --- | --- | --- | --- |
| Intercept | -2.401 | 0.346 | -6.935 | <0.001 |
| Area A11 | -0.002 | 0.433 | -0.004 | 0.996 |
| Area A10 | -0.391 | 0.486 | -0.805 | 0.422 |
| Area A7 | -0.358 | 0.503 | -0.712 | 0.477 |
| Area A6 | 0.179 | 0.464 | 0.387 | 0.699 |
| Area A5 | 0.653 | 0.450 | 1.452 | 0.148 |
| Area A4 | 0.337 | 0.419 | 0.805 | 0.422 |
| Area A3 | 0.332 | 0.385 | 0.862 | 0.389 |
| Area A2 | 0.865 | 0.422 | 2.049 | 0.042 |
| Area A1 | 0.542 | 0.370 | 1.462 | 0.145 |
| Dispersion parameter for quasibinomial family taken to be 3.516. | | | | |
| Null deviance: 931.18 on 269 degrees of freedom. | | | | |
| Residual deviance: 857.84 on 260 degrees of freedom. | | | | |
